# Job satisfaction, work commitment, and intention to leave among cardiac technologists in Saudi Arabia

**DOI:** 10.1016/j.pmedr.2025.103172

**Published:** 2025-07-12

**Authors:** Lamia Al Saikhan

**Affiliations:** Department of Cardiac Technology, College of Applied Medial Sciences, Imam Abdulrahman Bin Faisal University, Dammam 34212, Saudi Arabia

**Keywords:** Cardiac technologist, Work commitment, Healthcare, Intention to leave, And job satisfaction

## Abstract

**Objectives:**

We examined the factors influencing job satisfaction and work commitment among cardiac technologists across different healthcare settings, and the influence of these factors on the likelihood of leaving their current job.

**Methods:**

In this cross-sectional study, an online self-administered survey using a previously validated questionnaire was distributed from August to November 2022 to all graduates of the cardiac technology program at the *Imam* Abdulrahman bin Faisal University in the Eastern Province of Saudi Arabia between 2013 and 2022**.**

**Results:**

Of 204 graduates, 141 completed the survey(69.1 % response rate). Of these, 105(74.5 %) were employed during the survey and included in the final analysis. All respondents were women, and the majority(76.9 %) were aged 25–30 years, had a bachelor's degree(95.2 %), and were per-forming noninvasive procedures(echocardiography; 68.3 %). Most respondents worked eight to nine hours daily, and 56.6 % worked at government hospitals. Most respondents reported some level of job satisfaction with their current job(12.4 %, 35.2 %, and 25.7 % were extremely satisfied, satisfied, and slightly satisfied, respectively). However, 54 % of the respondents still intended to leave their current jobs. Their likelihood of leaving their current job was associated with place of practice(*p* = 0.01) and monthly income(*p* = 0.00). Place of practice, overnight call system, day/night shift system, and monthly income were associated with job satisfaction and work commitment(*p* < 0.05).

**Conclusions:**

The surveyed cardiac technologists were mostly satisfied with and relatively committed to their current jobs. However, they intended to leave. Future research exploring the reasons behind cardiac technologists' intentions to leave their cardiac technology practice jobs is needed.

## Highlights

• Job satisfaction and work commitment among cardiac technologists in Saudi Arabia.
• Over 50 % of cardiac technologists intend to leave despite high satisfaction.
• Place of practice and salary affect cardiac technologists' intention to leave jobs.
• Most respondents are women, aged 25–30, with a bachelor's in cardiac technology.

## 1 Introduction

Cardiac technology is an internationally recognized allied healthcare profession that focuses on diagnosing and managing patients with cardiovascular diseases (CVD). Cardiac technologists are highly skilled professionals qualified to provide patient care by performing a range of cardiovascular investigations, categorized as either non-invasive, such as echocardiography, or invasive, such as cardiac catheterization procedures, either diagnostically or through interventions. The overall employment of cardiac technologists in the United States is increasing faster than the average for other professions (estimated increase by 10 % from 2021 to 2031) (*J. Am. Soc. Echocardiogr.*, 2009; *Bureau of Labor Statistics, U.S. Department of Labor, Occupational Outlook Handbook, Medical Sonographers and Cardiovascular Technologists and Technicians*, 2025). In Saudi Arabia, the cardiac technology program was first established in 2008–2009 to address the scarcity of qualified cardiac sonographers and/or invasive cardiovascular specialists (*Program Overview, Cardiac Technology Department. Imam Abdulrahman bin Faisal University*, 2025), and to ensure that educational outputs were aligned with the projected labor market demands for specialized healthcare services (Vision 2030, n.d.).

Job satisfaction is multifactorial construct influenced by factors such as salary and benefits, quality of supervision/management, working environment, and working conditions (Blaauw et al., 2013). Further, job satisfaction is an important factor associated with employee turnover (Blaauw et al., 2013). While job satisfaction reflects an employee's contentment with their current job, work commitment refers to the psychological attachment and loyalty an individual feels towards their organization or profession—both of which independently influence turnover intentions (Meyer and Allen, 1991). Turnover intention, or intention to leave, is an increasingly recognized global challenge that threatens the function of healthcare sectors; therefore, adopting strategies to improve the retention rate of healthcare workers is essential to maintain a good quality healthcare system (Blaauw et al., 2013; Abera et al., 2014). Low pay, limited fringe benefits, and poor quality of supervision/management are among several factors that influence healthcare workers' decisions to continue (or discontinue) their employment (Almutairi and Idrus, 2010; Tourangeau et al., 2010). High turnover leads to adverse consequences, including the loss of experienced healthcare workers, short staffing, and reduced organizational performance (Blaauw et al., 2013; Tourangeau et al., 2010; Meier and Hicklin, 2008).

In Saudi Arabia, job satisfaction and its determining factors among various groups of healthcare professionals, including nurses, pharmacists, and physicians, have been previously reported (Almutairi and Idrus, 2010; Al-Muallem and Al-Surimi, 2019; Bawakid et al., 2017; Almalki et al., 2012; Kalantan Khalid et al., 1999; Alkassabi et al., 2018). However, as cardiac technology is an emerging allied healthcare profession in the Kingdom, no studies have been conducted on job satisfaction among Saudi Arabian cardiac technologists. Therefore, we aimed to investigate job satisfaction and commitment towards work among licensed cardiac technologists working in various healthcare settings, and the influence of these factors on the likelihood of employment discontinuation (i.e., intention to leave). We also aimed to explore the sociodemographic and occupational factors associated with job satisfaction among these cardiac technologists. Our findings will help identify potentially important factors affecting job satisfaction among cardiac technologists and provide recommendations for stakeholders to develop evidence-based retention policies for human health resources in Saudi Arabia. This is of value as the need for cardiac technology graduates is expected to increase for the aforementioned reasons.

## 2 Methods

### 2.1. Study population

2.1

This cross-sectional study was approved by the local Institutional Review Board of *Imam* Abdulrahman bin Faisal University (IAU) in the Eastern Province of Saudi Arabia (IRB-2022-03-234) and was conducted following the Declaration of Helsinki. All participants gave written informed consent prior to completing the questionnaire. This study included all alumni who graduated between 2013 and 2022 from the IAU Cardiac Technology Program. The program's alumni were deemed appropriate for the study as its educational outcomes aligned with the international qualifications of the profession. The program was exclusive to female students. Hence, all participants in this study were female. The survey was distributed electronically from August to November 2022 using Google Forms. Non-respondents received up to four reminder notifications. The invitation letter to the participants outlined the survey's purpose, included the investigators' contact information, and assured participants of the study's confidentiality. No personal identifying information was requested from any participant.

### 2.2 Survey

Established questionnaires in the literature were reviewed, and a validated tool based on Herzberg's theory of job satisfaction for measuring job satisfaction, commitment to work, and intention to leave among healthcare workers, (Al-Muallem and Al-Surimi, 2019; Chua et al., 2014) was used to develop the survey. The tool had been previously tested for validity and reliability in a Saudi context (Al-Muallem and Al-Surimi, 2019). Herzberg's theory of job satisfaction includes hygiene and motivational factors associated with preventing job dissatisfaction and increasing job satisfaction. The survey consisted of four sections: demographic characteristics, education/qualifications, current job features, and job satisfaction. The latter section consisted of subsections on job satisfaction and work commitment, comprising 15 statements, followed by items on overall job satisfaction and intention to leave the current position. The survey was assessed for content validity by two experts and amended if needed, and for face validity by piloting the survey among 10 participants for any ambiguity in content before being formally distributed. The Cronbach's alpha, an indicator for questionnaire reliability, was 0.88 for the job satisfaction and 0.70 for the work commitment scales. The amendments were made according to the received feedback to enhance the instrument's design, which was subsequently approved and distributed to all potential respondents.

### 2.3 Statistical analysis

For descriptive statistics, results were presented as mean ± SD or median (interquartile range) for continuous variables, and as counts (percentages) for categorical variables. Differences between two groups were assessed using the two-sample *t*-test or Wilcoxon rank-sum test for continuous variables and the χ^2^ test or Fisher exact test for categorical variables. Differences between more than two groups were assessed using analysis of variance or Kruskal–Wallis test. A *P*-value of <0.05 was considered statistically significant.

The required sample size was 139 participants determined using an online calculator based on a confidence level of 95 %, an error margin of 5 %, and a response distribution of 50 % (Raosoft Inc., Seattle, Washington, USA). The survey was distributed to 215 graduates.

STATA 15.1 was used to perform the analysis (StataCorp LLC, Houston, TX, USA).

## 3 Results

### 3.1 Study population

Eleven of 215 cardiac technology graduates were excluded from the study due to lost to follow-up. Of the remaining 204 participants, 141 completed the survey (69.1 % response rate). Of the 141 participants, 105 (74.5 %) were employed during the survey and included in the final analysis. Table 1 summarizes respondents' characteristics. All respondents (100 %) were women, and the majority (76.9 %) were aged between 25 and 30. Of these, 62.9 % were married, 95.2 % held a bachelor's degree, and 68.3 % were employed to perform noninvasive procedures. Of those, 83.2 % worked in the Eastern region of Saudi Arabia and 56.6 % worked at government hospitals. Most respondents worked eight to nine hours daily with less than one to three years of experience and a monthly income of 6001–10,000 Saudi riyals (∼1600–2670 United States dollars) or 10,001–15,000 Saudi riyals (∼2670–4000 United States dollars).

**Table 1:** Baseline characteristics of cardiac technologists in Saudi Arabia 2022 (*n* = 105).

Characteristics	n (%)
Female	105 (100)
Age (years)	
< 25	18 (17.3)
25–30	80 (76.9)
> 30	6 (5.8)
Marital status	
Single	39 (37.1)
Married	66 (62.9)
Location of residence	
Eastern region	95 (90.5)
Northern region	0 (0)
Western (Mecca/Jeddah) region	2 (1.9)
Southern (Asir) region	0 (0)
Central (Riyadh) region	7 (6.7)
Other	1 (0.95)
Sub-specialty	
Invasive: Cardiac catheterization	33 (31.7)
Non-invasive: Echocardiography	71 (68.3)
Highest qualification	
Bachelor's degree	100 (95.2)
Master's degree	4 (3.8)
Doctorate degree	1 (0.95)
Location of current job	
Eastern region	84 (83.2)
Northern region	0 (0)
Western (Mecca/Jeddah) region	8 (7.9)
Southern (Asir) region	0 (0)
Central (Riyadh) region	9 (8.9)
Number of years in practice	
< 1 year	31 (30.7)
1–3 years	42 (41.6)
> 3–5 years	15 (14.8)
> 5–7 years	11 (10.9)
> 7 years	2 (2.0)
Place of practice	
Government/public hospital	56 (56.6)
Private hospital	33 (33.3)
Private clinic	3 (3.0)
Industry	1 (1.0)
Academia/university	6 (6.1)
Monthly income (in Saudi riyal)	
≤ 6000 (≤ ∼1600, USD)	12 (12.0)
6001–10,000 (∼1600–2670, USD)	42 (42.0)
10,001–15,000 (∼2670–4000, USD)	41 (41.0)
≥ 15,001 (≥ ∼4000, USD)	3 (5.0)
Average working hours per day	
7	3 (3.0)
8	46 (45.5)
9	46 (45.5)
10	2 (2.0)
Other	4 (4.0)

USD, United States Dollar.

### 3.2 Job satisfaction

Table 2 summarizes cardiac technologists' responses across all job satisfaction parameters. Approximately 70 % of the respondents reported being satisfied with their job; feeling happy to go to work daily; describing their job to family and friends as a great opportunity that allowed them to utilize their abilities; indicating having enough flexibility to choose how to perform their tasks; feeling free to exercise their judgement in their work; being satisfied at the end of each day, believing the day had been well spent; and feeling fortunate to have their job. Furthermore, 82 % of the respondents reported feeling a sense of accomplishment in their work. However, 44 % were unsatisfied with their salary, 46 % expressed dissatisfaction with the fringe benefits provided by their current job, 45 % were unsatisfied with the personnel policies of their organization, and 40 % were unsatisfied with their style and quality of supervision.

**Table 2:** Job satisfaction among cardiac technologists in Saudi Arabia 2022 (n = 105).

Statement	Strongly disagree *n* (%)	Disagree *n* (%)	Slightly disagree *n* (%)	Slightly agree *n* (%)	Agree *n* (%)	Strongly agree *n* (%)	Mean score (SD)
“I look forward to coming to work every day.”	6 (5.7)	11 (10.5)	13 (12.4)	28 (26.7)	37 (35.2)	10 (9.5)	4.04 (1.34)
“I talk about my job with my family and my friends because it is a great job.”	5 (4.8)	14 (13.3)	15 (14.3)	27 (25.7)	31 (29.5)	13 (12.4)	3.99 (1.38)
“My job provides me with a broad opportunity to use my abilities.”	5 (4.8)	13 (12.4)	11 (10.5)	17 (16.2)	43 (41.0)	16 (15.2)	4.22 (1.42)
“I have sufficient freedom to use my own judgement in my job.”	3 (2.9)	14 (13.3)	15 (14.3)	23 (21.9)	39 (37.1)	11 (10.5)	4.09 (1.32)
“My job provides me enough flexibility to choose any method of doing the job.”	4 (3.8)	20 (19.1)	13 (12.4)	24 (22.9)	32 (30.5)	12 (11.4)	3.91 (1.41)
“I get a feeling of accomplishment from my job.”	3 (2.9)	7 (6.7)	9 (8.6)	33 (31.4)	41 (39.1)	12 (11.4)	4.31 (1.17)
“At the end of each working day, I feel that the day has been well-spent.”	8 (7.6)	9 (8.6)	13 (12.4)	28 (26.7)	37 (35.2)	10 (9.5)	4.02 (1.38)
“If I were to start my career again, I would choose this job.”	12 (11.4)	16 (15.2)	15 (14.3)	15 (14.3)	32 (30.5)	15 (14.3)	3.80 (1.63)
“Other people would be very lucky to get a job like mine.”	6 (5.7)	13 (12.4)	9 (8.6)	22 (21.0)	38 (36.2)	17 (16.2)	4.18 (1.45)
“I am satisfied with my job.”	7 (6.7)	11 (10.5)	11 (10.5)	28 (26.7)	32 (30.5)	16 (15.2)	4.09 (1.43)
“I am satisfied with my salary.”	18 (17.1)	15 (14.3)	13 (12.4)	21 (20.0)	31 (29.5)	7 (6.7)	3.50 (1.61)
“I am satisfied with the fringe benefits offered by my job.”	13 (12.4)	16 (15.2)	19 (18.1)	25 (23.8)	25 (23.8)	7 (6.7)	3.51 (1.48)
“I am satisfied with the working conditions.”	8 (7.6)	18 (17.1)	17 (16.2)	22 (21.0)	33 (31.4)	7 (6.7)	3.71 (1.43)
“I am satisfied with the personnel policies of this organization.”	10 (9.5)	19 (18.1)	18 (17.1)	18 (17.1)	31 (29.5)	9 (8.6)	3.65 (1.51)
“I am satisfied with the style and quality of supervision.”	17 (16.2)	15 (14.3)	10 (9.5)	23 (21.9)	31 (29.5)	9 (8.6)	3.60 (1.62)

SD, standard deviation.

### 3.3 Commitment to work

Table 3 summarizes the responses of the cardiac technologists across all parameters of commitment to work. Approximately 90 % of the respondents expressed a willingness to put in extra effort beyond what is typically expected to help their workplace succeed. More than 70 % reported feeling proud to tell others they were part of their organization, were very glad they chose to work there, and deeply cared about its fate. However, more than 70 % indicated that they could easily work for a different organization. About 60 % of the respondents reported that their values aligned with those of their organization, described their workplace to family and friends as an excellent organization to work for, and felt that their workplace inspired their job performance. Furthermore, approximately 86 % of the respondents disagreed with the statement that choosing to work for their organization was a definite mistake, and 54 % stated that their organization was the best possible place to work. Nearly 50 % of the respondents did not feel loyal towards their organizations; they believed there was little to be gained by staying with their organization and found it difficult to agree with the organizations' policies regarding employee-related matters. Forty-three percent of respondents agreed that only minimal changes in their current circumstances would be enough to motivate them to leave their organization.

**Table 3:** Commitment to work among cardiac technologists in Saudi Arabia 2022 (n = 105).

Statement	Strongly disagree *n* (%)	Disagree *n* (%)	Slightly disagree *n* (%)	Slightly agree *n* (%)	Agree *n* (%)	Strongly agree *n* (%)	Mean score (SD)
“I am willing to put in effort beyond what is normally expected to help my workplace be successful.”	1 (0.9)	5 (4.8)	5 (4.8)	18 (17.1)	46 (43.8)	30 (28.6)	4.84 (1.10)
“I talk about my workplace to my friends because it is a great organization to work for.”	8 (7.6)	18 (17.1)	13 (12.4)	28 (26.7)	30 (28.6)	8 (7.6)	3.74 (1.43)
“I feel very little loyalty for this organization.”	11 (10.5)	22 (21.0)	14 (13.3)	24 (22.9)	28 (26.8)	6 (5.7)	3.51 (1.48)
“I would accept almost any type of job assignment to keep working at this organization.”	16 (15.2)	29 (27.6)	21 (20.0)	21 (20.0)	14 (13.3)	4 (3.8)	3.0 (1.41)
“I find that my values and those of my organization are very similar.”	13 (12.4)	19 (18.1)	12 (11.4)	22 (21.0)	33 (31.4)	6 (5.7)	3.58 (1.53)
“I am proud to tell others that I am a part of this organization.”	6 (5.7)	8 (7.6)	9 (8.6)	23 (21.9)	40 (38.1)	19 (18.1)	4.33 (1.38)
“I could just as well be working for a different organization.”	2 (1.9)	14 (13.3)	10 (9.5)	20 (19.1)	47 (44.8)	12 (11.4)	4.26 (1.29)
“My workplace inspires me to do my best job.”	7 (6.7)	21 (20.0)	15 (14.3)	24 (22.9)	31 (29.5)	7 (6.7)	3.69 (1.42)
“It would take very little change in my present circumstances to make me leave this organization.”	6 (5.7)	26 (24.8)	28 (26.7)	22 (21.0)	17 (16.2)	6 (5.7)	3.34 (1.32)
“I am extremely glad that I choose this organization to work for.”	6 (5.7)	9 (8.6)	13 (12.4)	27 (25.7)	36 (34.3)	14 (13.3)	4.14 (1.36)
“There is not much to be gained by sticking with this organization.”	9 (8.6)	24 (22.9)	17 (16.2)	23 (21.9)	22 (21.0)	10 (9.5)	3.52 (1.49)
“I find it often difficult to agree with my organization's policies on important matters related to its employees.”	13 (12.4)	21 (20.0)	16 (15.2)	24 (22.9)	24 (22.9)	7 (6.7)	3.44 (1.51)
“I really care about the fate of this organization.”	4 (3.8)	7 (6.7)	20 (19.1)	23 (21.9)	42 (40.0)	9 (8.6)	4.13 (1.24)
“For me, this is the best possible organization to work for.”	9 (8.6)	26 (24.8)	13 (12.4)	20 (19.1)	27 (25.7)	10 (9.5)	3.57 (1.54)
“Deciding to work for this organization was a definite mistake on my part.”	36 (34.3)	41 (39.1)	13 (12.4)	9 (8.6)	3 (2.9)	3 (2.9)	2.15 (1.23)

SD, standard deviation.

### 3.4 Overall satisfaction and intention to leave

Table 4 summarizes the overall cardiac technologists' satisfaction and intention to leave. Most cardiac technologists indicated that they were satisfied with their current jobs (12.4 %, 35.2 %, and 25.7 % were extremely satisfied, satisfied, and slightly satisfied, respectively), whereas only 6.7 % were extremely dissatisfied. However, 54 % of the cardiac technologists stated that they intended to leave their current job for any reason, with the remainder stating that they were unlikely to leave.

**Table 4:** Overall job satisfaction and intentions to leave among cardiac technologists in Saudi Arabia 2022 (n = 105).

Overall satisfaction with the current job	n (%)
*How satisfied are you with your current job?*	
Extremely dissatisfied	7 (6.7)
Dissatisfied	10 (9.5)
Slightly dissatisfied	11 (10.5)
Slightly satisfied	27 (25.7)
Satisfied	37 (35.2)
Extremely satisfied	13 (12.4)
Intention to leave the current job	

*How likely are you to leave your current job for any reason?*	
Very unlikely	16 (15.2)
Unlikely	32 (30.5)
Likely	37 (35.2)
Very likely	20 (19.1)

### 3.5 Factors associated with the cardiac technologists job satisfaction, commitment to work, and likelihood to remain in current job

Table 5 summarizes the associations between cardiac technologists' baseline characteristics and the likelihood of continuing with their current jobs. The cardiac technologists' likelihood to continue in their current job was associated with their monthly income (*p* = 0.00) and place of practice (*p* = 0.01). When working in healthcare settings, the overnight call and day/night shift systems were variables that tended to be associated with cardiac technologists' likelihood of continuing with their current job. However, these differences did not reach statistical significance (*p* = 0.07 and p = 0.07, respectively).

**Table 5:** Association between baseline characteristics of cardiac technologists in Saudi Arabia 2022 and likelihood to continue in their current job.

**Characteristics**	**Likely to continue** **(*n* = 48), *n* (%)**	**Unlikely to continue** **(*n* = 57), *n* (%)**	***P* value**
Age (years)			*0.19*
< 25	40 (85.1)	40 (70.2)	
25–30	5 (10.6)	13 (22.8)	
> 30	2 (4.3)	4 (7.0)	
Marital status			*0.25*
Single	15 (31.2)	24 (42.1)	
Married	33 (68.8)	33 (57.9)	
Subspeciality			*0.70*
Cardiac catheterization	14 (29.8)	19 (33.3)	
Echocardiography	33 (70.2)	38 (66.7)	
Monthly income (in Saudi riyal)			*0.00*
≤ 6000 (≤ ∼1600, USD)	4 (8.7)	8 (14.8)	
6001–10,000 (∼1600–2670, USD)	12 (26.1)	30 (55.6)	
10,001–15,000 (∼2670–4000, USD)	28 (60.9)	13 (24.1)	
≥ 15,001 (≥ ∼4000, USD)	2 (4.3)	3 (5.6)	
Highest level of education			*0.54*
Bachelor's degree	45 (93.8)	55 (96.5)	
Master's degree	2 (4.2)	2 (3.5)	
Doctorate degree	1 (2.0)	0 (0)	
Average working hours per day			*0.80*
7	2 (4.3)	1 (1.8)	
8	20 (42.6)	26 (48.2)	
9	23 (48.9)	23 (42.6)	
10	1 (2.1)	1 (1.8)	
Others	1 (2.1)	3 (5.6)	
Place of practice (current job)			*0.01*
Government/public hospital	32 (69.6)	24 (45.3)	
Private hospital	9 (19.6)	24 (45.3)	
Private clinic	0 (0)	3 (5.7)	
Industry	0 (0)	1 (1.9)	
Academia/university	5 (10.9)	1 (1.9)	
Number of years in practice (current job)			*0.46*
< 1	13 (27.7)	18 (33.3)	
1–3	17 (36.2)	25 (46.3)	
> 3–5	10 (21.3)	5 (9.3)	
> 5–7	6 (12.8)	5 (9.3)	
> 7	1 (2.1)	(1.9)	
Overnight call system if working in healthcare settings			*0.07*
Yes	19 (41.3)	33 (61.1)	
No	21 (45.7)	19 (35.2)	
N/A	6 (13.0)	2 (3.7)	
Day/night shift system if working in healthcare settings			*0.07*
Yes	9 (19.2)	20 (37.7)	
No	33 (70.2)	31 (58.5)	
N/A	5 (10.6)	2 (3.8)	

USD, United States Dollar. Statistical analysis was performed using Chi-square test.

Table 6 summarizes the associations among cardiac technologists' baseline characteristics, job satisfaction, and work commitment. Monthly income, place of practice, overnight call system (if working in a healthcare setting), and day/night shift system (if working in a healthcare setting) were significantly linked to job satisfaction and work commitment (*p* < 0.05).

**Table 6:** Association between baseline characteristics of cardiac technologists in Saudi Arabia 2022 and their job satisfaction and work commitment (n = 105).

Characteristics	Job satisfaction Median (IQR)	P value	Work commitment Median (IQR)	P value
Age (years)		*0.65*		*0.09*
< 25	60.5 (50.5–71)		57 (52–61)	
25–30	55 (45–68)		53 (44–55)	
> 30	50.5 (42–80)		53.5 (52–57)	
Marital status		*0.68*		*0.21*
Single	60 (47–73)		53 (49–60)	
Married	58 (48–70)		56 (52–61)	
Subspeciality		*0.24*		*0.61*
Cardiac catheterization	61 (51–73)		57 (52–60)	
Echocardiography	58 (47–70)		54 (50–61)	
Monthly income (in Saudi riyal)		*0.00*		*0.00*
≤ 6000 (≤ ∼1600, USD)	44 (35.5–54)		51.5 (45.5–53.5)	
6001–10,000 (∼1600–2670, USD)	57 (45–68)		54 (51–60)	
10,001–15,000 (∼2670–4000, USD)	66 (54–75)		57 (53–62)	
≥ 15,001 (≥ ∼4000, USD)	73 (64–73)		61 (59–65)	
Highest level of education		*0.27*		*0.50*
Bachelor's degree	58 (47.5–69.5)		55 (51–60)	
Master's degree	75 (54–81.5)		60 (52–61.5)	
Doctorate degree	73 (73–73)		61 (61–61)	
Average working hours per day		*0.21*		*0.13*
7	73 (61–86)		61 (61–74)	
8	57.5 (49–70)		54 (50–61)	
9	60.5 (51–72)		56.5 (52–60)	
10	55.5 (38–73)		58.5 (58–59)	
Others	29 (24.5–54.5)		48.5 (37–57)	
Place of practice (current job)		*0.00*		*0.01*
Government/public hospital	62.5 (52–73)		57 (51.5–61.5)	
Private hospital	54 (42–58)		53 (51–57)	
Private clinic	44 (39–47)		44 (43–54)	
Industry	60 (60–60)		56 (56–56)	
Academia/university	74 (61–86)		61 (61–67)	
Years of practice (current job)		*0.66*		*0.13*
< 1	58 (44–73)		54 (48–59)	
1–3	57.5 (46–69)		53.5 (51–61)	
> 3–5	60 (50–73)		61 (51–65)	
> 5–7	60 (51–78)		57 (55–57)	
> 7	68.5 (64–73)		63 (61–65)	
Overnight call system if working in healthcare settings		*0.04*		*0.03*
Yes	56 (45.5–68)		54 (48.5–59)	
No	59 (51.5–73)		55 (52.5–60.5)	
N/A	71.5 (63–76)		61 (59.5–63.5)	
Day/night shift system if working in healthcare settings		*0.00*		*0.00*
Yes	47 (39–56)		51 (45–54)	
No	62 (54–73)		57 (53–61.5)	
N/A	75 (61–86)		61 (61–67)	

IQR, interquartile range. USD, United States Dollar. Statistical analysis was performed using the Kruskal-Wallis test or Wilcoxon rank-sum test.

## 4 Discussion

This study examined job satisfaction, work commitment, and turnover intentions among cardiac technologists working in Saudi Arabia. We found that the job satisfaction of cardiac technologists varied moderately across all job satisfaction parameters. Various factors impacted their job satisfaction, including salary and benefits, the quality of supervision and management, and the overall working environment and conditions. Our findings were consistent with those of previous studies on job satisfaction among healthcare workers in other medical fields (Al-Muallem and Al-Surimi, 2019; Chua et al., 2014). While job satisfaction was high among the surveyed cardiac technologists, their intention to quit their current roles was also relatively high. This aligns with the findings of Al-Muallem et al. (2019) who investigated job satisfaction, work commitment, and turnover intentions among 325 pharmacists in Saudi Arabia. The study found that despite being satisfied and committed to their roles, pharmacists still had a strong desire to leave (Al-Muallem and Al-Surimi, 2019). As job satisfaction increases, the likelihood of employee absenteeism and consequently turnover intention decreases (Aziri, 2011). Nevertheless, our findings suggest that job satisfaction among cardiac technologists does not necessarily exclude the possibility of them leaving their current position. Besides job satisfaction, factors such as motivation, workload, interpersonal relationships, career development opportunities, and organizational policies and/or commitment are closely linked to turnover intention among healthcare workers (Bonenberger et al., 2014).

In this study, most cardiac technologists expressed commitment towards their work, demonstrating qualities such as caring about the organization's fate, being grateful for choosing their organization to work for, and being proud to express that they were part of their organization (work environment). However, most cardiac technologists indicated that they could just as easily work for another organization. Our findings align with those of previous studies, which also indicated that the extent of employees' commitment to work is influenced by leadership behaviour (Al-Muallem and Al-Surimi, 2019; Chua et al., 2014; Yousef, 2000). A consultative/participative leadership style is associated with higher organizational commitment as well as higher job satisfaction and employee performance (Chua et al., 2014; Yousef, 2000). Employee empowerment positively impacts organizational trust and commitment (Laschinger et al., 2001). Therefore, an employee's willingness to continue in or leave their current job is influenced by their organizational commitment. This aligns with previous observations that overall job satisfaction and commitment play a significant role in turnover rates (Camp, 1994).

While the surveyed Saudi cardiac technologists were largely satisfied with and relatively committed to their current jobs, the finding that 54 % expressed an intention to leave is concerning and highlights potential challenges for workforce stability. This aligns with a previous work by Al-Muallem et al. (2019), who found that high job satisfaction did not necessarily mitigate turnover intentions among healthcare professionals in Saudi Arabia (Al-Muallem and Al-Surimi, 2019). In our study, respondents' intentions to continue or leave their jobs were significantly associated with monthly income and place of practice. All respondents were women, and their intention to remain was often influenced by the job security offered in the public sector. Similar trends have been observed elsewhere; for instance, Chua et al. (2014) reported that female healthcare workers in Malaysia were two and a half times less likely to leave public sector positions compared to males (Chua et al., 2014). Al-Muallem et al. (2019) reported that place of practice, current position and monthly income were significant factors affecting the likelihood of healthcare workers in Saudi Arabia continuing their current employment (Al-Muallem and Al-Surimi, 2019), which is consistent with our findings. Low monthly income has been identified as a primary predictor of turnover intention among healthcare workers (Almutairi and Idrus, 2010; Tourangeau et al., 2010; Mott, 2000; Chan and Morrison, 2000). Furthermore, factors such as overnight call systems and shift patterns were significantly linked to job satisfaction and work commitment in our cohort, reflecting previous studies that underscore the impact of income, working conditions, and workload on employee retention (Abera et al., 2014; Tourangeau et al., 2010; Al-Muallem and Al-Surimi, 2019; Kalantan Khalid et al., 1999; A S., 2015).

CVD is among the leading causes of death in Saudi Arabia (Collaborators, 2020; *WHO. Saudi Arabia. World Health Organization – noncommunicable Diseases (NCD) Country Profiles*, 2018). Cardiovascular risk factors contribute to the disability burden among the population of Saudi Arabia (Collaborators, 2020; Moradi-Lakeh et al., 2016; Moradi-Lakeh et al., 2017; El Bcheraoui et al., 2014; Memish et al., 2014; Moradi-Lakeh et al., 2015). Therefore, the demand for healthcare services, especially specialized cardiac care, is anticipated to rise steadily. This study identified potentially important issues with cardiac technologists' job satisfaction and work commitment and provided stakeholders with evidence on the associated factors and concerns and their influence on the likelihood of cardiac technologists leaving their current employer. Our findings highlight the need to develop an evidence-based retention policy for healthcare professionals, particularly cardiac technologists, to meet projected labour market needs and avoid potential adverse consequences associated with a high turnover rate (Blaauw et al., 2013; Tourangeau et al., 2010; Meier and Hicklin, 2008). These baseline workforce data can serve as a reference for comparing future cohorts and provide valuable insights for planning and developing the cardiac technology workforce.

This study has some limitations that must be acknowledged. There was a potential bias due to non-response errors caused by participants' inability, unavailability, or unwillingness to participate. This led to the exclusion of some participants, and potential differences between those excluded and those included could not be ruled out. However, multiple follow-ups and notifications were attempted with non-respondents to maximize the response rate while minimizing bias. The study sample size is relatively small, and its external validity may be limited, as the studied sample primarily included female cardiac technologists working in the Eastern province of Saudi Arabia. Hence, our findings may not be generalizable to male cardiac technologists or those from other regions of the country. Nevertheless, our study offers valuable preliminary data that can inform future research.

## 5 Conclusions

The study revealed varying levels of job satisfaction and work commitment among cardiac technologists working in different healthcare settings in Saudi Arabia. Although most cardiac technologists reported satisfaction and commitment to their current roles, a majority still expressed an intention to leave. Notably, factors such as monthly income, place of practice, overnight call duties, and day/night shift schedules were significantly associated with job satisfaction and work commitment. Future research exploring the factors driving cardiac technologists' intentions to leave their cardiac technology practice jobs is needed.

## Authorship contribution

LA conceived and designed the study, performed the data collection, statistical analysis and data interpretation. LA drafted the manuscript and critically reviewed, amended and approved the manuscript.

## CRediT authorship contribution statement

**Lamia Al Saikhan:** Writing – review & editing, Writing – original draft, Methodology, Formal analysis, Data curation, Conceptualization.

## Funding

None.

## Declaration of competing interest

The authors declare that they have no known competing financial interests or personal relationships that could have appeared to influence the work reported in this paper.

## Data Availability

Data will be made available on request.
